# Self-consistent dielectric functions of materials: Toward accurate computation of Casimir–van der Waals forces

**DOI:** 10.1126/sciadv.abg2272

**Published:** 2021-05-26

**Authors:** Mohsen Moazzami Gudarzi, Seyed Hamed Aboutalebi

**Affiliations:** 1 National Graphene Institute, University of Manchester, Manchester, UK.; 2 Condensed Matter National Laboratory, Institute for Research in Fundamental Sciences, Tehran 19395-5531, Iran.

## Abstract

Research on theoretical calculation of Casimir–van der Waals (vdW) forces is characterized by a great number of inconsistencies and conflicting reports with widely differing results for many known materials, including water, contradicting experimental measurements. Despite its importance for conceptual advances in both fundamental aspects and practical applications, a universal framework for the accurate determination of Casimir-vdW forces is lacking. Here, we propose a universal theoretical platform for computing Casimir-vdW forces, accounting for the electronic dielectric constant, optical bandgap, density, and chemical composition. Using this methodology, we determine the dielectric function for 55 materials, over a wide range of photon energies, covering an extensive list of common metals, organic and inorganic semiconductors, and insulators. Internal consistency of the compiled data is validated using optical sum rules and Kramers-Kronig relations. We demonstrate that the calculated vdW forces based on these data match remarkably well with the experimentally measured vdW forces.

## Teaser

Accurate determination of dielectric function in the vacuum UV range enables the precise computation of Casimir-vdW forces.

## INTRODUCTION

In 1948, Casimir (*1*) theorized that quantum electromagnetic fluctuations or zero-point energy alterations result in attractive forces acting between perfect parallel neutral conductors in vacuum. Later, Lifshitz and his colleagues (*2*) devised a general theory for Casimir–van der Waals (vdW) forces, making it possible to calculate these forces from the complex dielectric function of materials. However, to this date, despite the importance of these forces in physical phenomena, such as wetting and adhesion (*3*–*5*), friction and polymer flow (*6*), (bio)colloidal stability (*7*, *8*), supramolecular chemistry (*9*, *10*), biophysiochemical interactions (*11*), protein folding, stability, and deformation (*12*), and even potency of anesthetics (*13*), many inconsistencies and contradicting results have been reported in the literature, making it difficult to tailor Casimir-vdW forces to practical applications.

For instance, the dielectric function of water has been repeatedly “improved,” with some early estimations proved to be inaccurate, yet still used by many researchers (*14*–*18*). Another key example is the case of ethanol, for which the dielectric function was calculated using wrong parameters leading to an absurd estimation of the infrared (IR) refractive indices—much higher than the indices reported for silicon (*19*, *20*)! Similar inaccuracy has marred the estimation of Teflon’s dielectric function (*20*). For less studied materials, the errors in the estimation of vdW interactions are even more drastic. As an example, the approximated Hamaker constant of graphene oxide across water varies between 2.4 and 49 zJ. (*21*, *22*). The usual error here originates from inaccurate estimation of the dielectric function based on a single harmonic oscillator to account for absorption bands in the ultraviolet (UV) region of light (*23*–*25*).

Here, we propose a theoretical framework for computing dielectric functions over the full frequency range necessary for the calculation of Casimir-vdW forces for 55 materials, covering various metals, semiconductors, and insulators, both organic and inorganic. Internal consistency of the results is evaluated using the optical sum rules and Kramers-Kronig (K-K) relations. We show that our as-calculated vdW forces between materials are in remarkable agreement with the experimental values reported over the span of the past half-century. Furthermore, we propose an improved empirical equation to model the electronic part of the dielectric function for semiconductors and insulators solely based on the material’s electronic dielectric constant, optical bandgap, density, and chemical composition.

## RESULTS

**Fig. 1:**
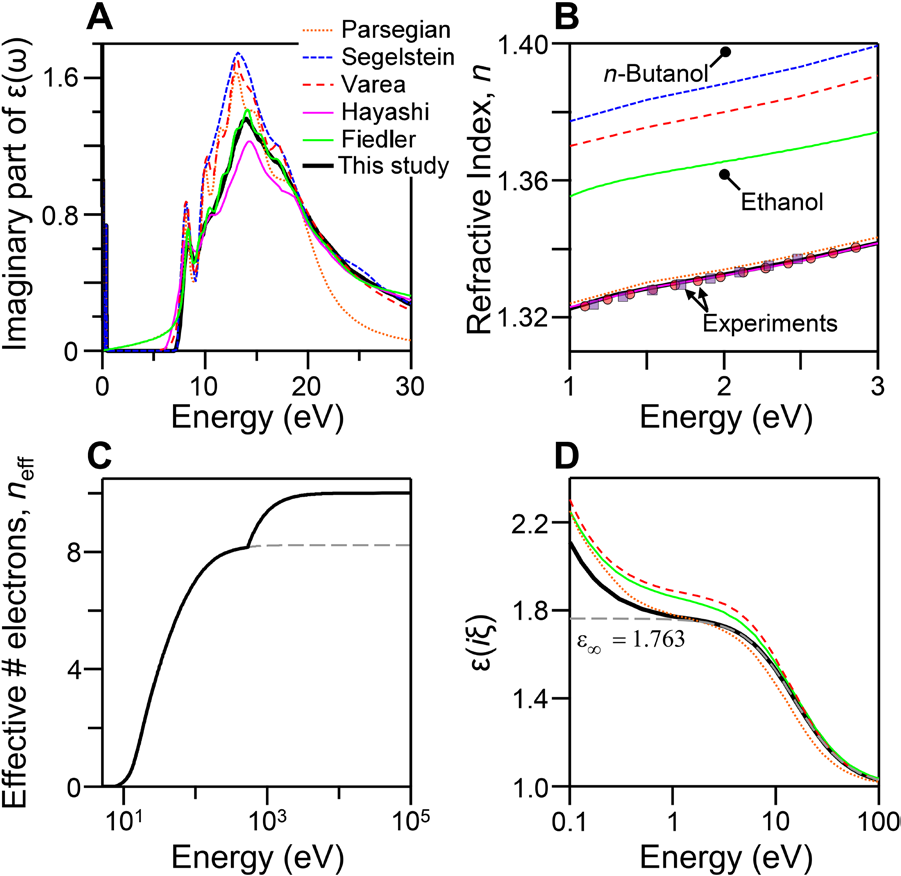
The dielectric function of water. (**A**) The imaginary part of the dielectric function of water in the VUV region reported by several groups (*14*, *15*, *17*, *18*, *30*). (**B**) The refractive indices of water in the visible region calculated using the K-K analysis of the optical data are presented in (A). Experimentally measured refractive indices of water reported by Kedenburg *et al*. (*29*) and Daimon and Masumura (*28*) are shown in (B). The refractive indices of ethanol and butanol are also shown here to demonstrate the magnitude of the error in the estimation of the dielectric function of water. (**C**) The effective number of electrons in the system as a function of photon energy. (**D**) The dielectric function of water at imaginary frequencies derived from the K-K transformation of data in (A).

We start with water and describe the sources of inconsistencies leading to conflicting results in the as-reported estimated dielectric functions. Unexpectedly, despite being of great importance, reliable data on the dielectric function of water in the vacuum UV (VUV) region are lacking. In the older literature, most studies cite the work of Heller *et al*. (*26*), where they measured reflectivity of water in the VUV region and obtained the complex refractive index of water. Hayashi *et al*. (*16*, *17*) later used inelastic x-ray scattering of water to estimate its dielectric function in a wide energy window. Figure 1A shows the imaginary part of the dielectric function (ε_2_) of water as reported in several previous studies. The largest variation among these datasets is in the magnitude of ε_2_, between 10 and 20 eV. The accuracy of each dataset is first evaluated by using the K-K analysis, which can interrelate the real and the imaginary parts of the dielectric function (please see section S1 and figs. S1 and S4 for more details). It should be noted that a self-consistent dataset for the dielectric function should follow sum rules and provide valid physical constants for materials (*27*). Although these procedures are widely used in the optics community, evaluating the self-consistency of dielectric functions used to calculate Casimir-vdW forces is not yet reported. Using the K-K analysis, we computed the refractive indices of water in the visible region and compared them to indices that were independently measured with an accuracy of better than 0.01% (*28*, *29*). Three of these six sets of data led to erroneous values for refractive indices of water in the visible region (Fig. 1B). Among them, the deviation observed for the data presented by Fiedler *et al*. (*18*) originates from the overestimation of ε_2_ below the bandgap of water. The model proposed by Parsegian (*30*) predicts accurate values for the refractive indices, but only due to the underestimation of ε_2_ at photon energies above 20 eV. Consequently, the data by Parsegian fail to account for the correct effective number of electrons in the system. These limitations have already been pointed out by Dagastine *et al*. (*31*).

At photon energies of about 14 eV, where there is large discrepancy among the analyzed sets of data, the absorption cross section of water is 1.9 ± 0.2 × 10^−17^ cm^2^ molecule^−1^ (*32*). This is equivalent to the extinction coefficient of 0.45 ± 0.05 and is in agreement with reports of Hayashi *et al*. (*16*, *17*) (*k* = 0.49 ± 0.02), but smaller than the one reported by Heller *et al*. (*26*) (*k* = 0.64). The earlier measurement by Hayashi *et al*. (*17*), however, overestimates ε_2_ at above 28 eV compared to their later reports and also to the relevant data tabulated in the The Center for X-ray Optics database (*33*). Here, we use the latest measurement of Hayashi *et al*. (*16*) to construct the dielectric function of water (see the “H_2_O” section in the Supplementary Materials for more details) because it is consistent with the tabulated values for the absorption cross section and x-ray refractive indices of water (*32*, *33*). Extrapolating these data to high photon energies using the available refractive indices in the x-ray region, the number of electrons in the system is found to be 10, equal to the nominal value for water (Fig. 1C). The current dataset is consistent with the accurately measured refractive indices in the visible region for liquid water (*28*, *29*) and correctly accounts for the number of electrons in water. The dielectric function at imaginary frequencies, required for Casimir-vdW force calculations, is shown in Fig. 1D, along with three other previous estimations.

Similar inconsistencies can be found in the optical data used to construct the dielectric functions of other materials. While there are numerous databases and handbooks available on optical constants of materials (*32*–*35*), compiling a self-consistent dataset is not necessarily straightforward. In the case of semiconductors and insulators, usually dielectric constants and refractive indices in the IR and visible regions are reported with high precision. In the VUV region, however, there are frequent inconsistencies, often not in the position of absorption bands but rather in their strength, as demonstrated above in the case of water. It should be noted that, although it is easy to measure complex dielectric functions of insulators in the visible and near-UV range, the techniques needed to measure optical constants of materials in the VUV range are at their infancy due to the high absorbance of almost all materials in this range (*36*). Nonetheless, the optical constants in the VUV region have the largest contribution to Casimir-vdW forces in the most relevant interbody distances, typically ranging from a few nanometers to a fraction of a micrometer (*30*). Therefore, it is a common practice to model these bands with harmonic oscillators.

Any dielectric function can be approximated with a finite number of these oscillators. The dielectric constant at any frequency ν is given by Eq. 1 (*30*)

$$\varepsilon (\nu )-1=\sum_{j,\nu \leq \omega_{\mathrm{Tj}}}^{\infty }{\left(\frac{\omega_{\mathrm{pj}}}{\omega_{\mathrm{Tj}}}\right)}^{2}$$ (1)

where ω*_p_* and ω*_T_* are the plasma and resonance frequencies of the harmonic oscillators. Plasma frequency is defined as

$$\omega_{p}=\sqrt{\frac{{\mathrm{ne}}^{2}}{\varepsilon_{0}m}}$$ (2)

where *n*, *e*, ε_0_, and *m* are the concentration of the oscillators (or carriers), elementary charge, vacuum permittivity, and the effective mass of carriers, respectively. Also, the effective number of electrons, *n*_eff_, in the system up to any frequency, ν, is given by

$$n_{\text{eff}}(\nu )=\frac{M_{w}\varepsilon_{0}m_{e}}{\rho N_{A}e^{2}\hbar^{2}}\sum_{0}^{\nu \geq \omega_{\mathrm{pj}}}{\omega_{p}}_{j}^{2}$$ (3)

where *M_w_*, *m_e_*, ρ, *N_A_*, and ℏ are the molecular mass, mass of electron, mass density, Avogadro number, elementary charge, and the Planck constant, respectively. Therefore, for any given absorption frequency, the oscillators’ strengths should be such that they satisfy the above sum rules, providing a basis for compiling materials’ optical data. Although these principles have been known for decades, dielectric functions commonly used for calculating Casimir-vdW forces are not usually derived from optical data conforming to the sum rules, as it was shown in the case of water.

In addition to water, we compiled and analyzed optical constants of 54 more materials and assessed the self-consistency of these data following the same procedure (figs. S9 to S63). Additional causality tests further proved the internal consistency of these data (fig. S4) (*37*). All these datasets predict the effective number of electrons in the systems correctly. Moreover, from the K-K analysis, one can calculate the refractive indices of these materials within the experimental error (fig. S1). Dielectric functions of many widely used materials such as silica, graphite, some organic solvents, and polymers are revisited to satisfy the above sum rules. The reviewed and compiled data are used to derive an empirical model to describe the dielectric functions of materials based on their widely available physical properties.

### Introducing the modified harmonic oscillator model

As we mentioned earlier, calculating Casimir-vdW forces requires optical constants in the range from zero to the soft x-ray region. However, based on Lifshitz’s theory, the dielectric function at imaginary frequencies, ε(*i*ξ), is sufficient for force calculation (*2*). The advantage of using dielectric functions at imaginary frequencies over real frequencies is the disappearance of resonance in the optical response of materials. Therefore, the dielectric function becomes a monotonically decaying function, reaching the static dielectric constant at zero frequency and decaying to one at large photon energies (*30*).

**Fig. 2:**
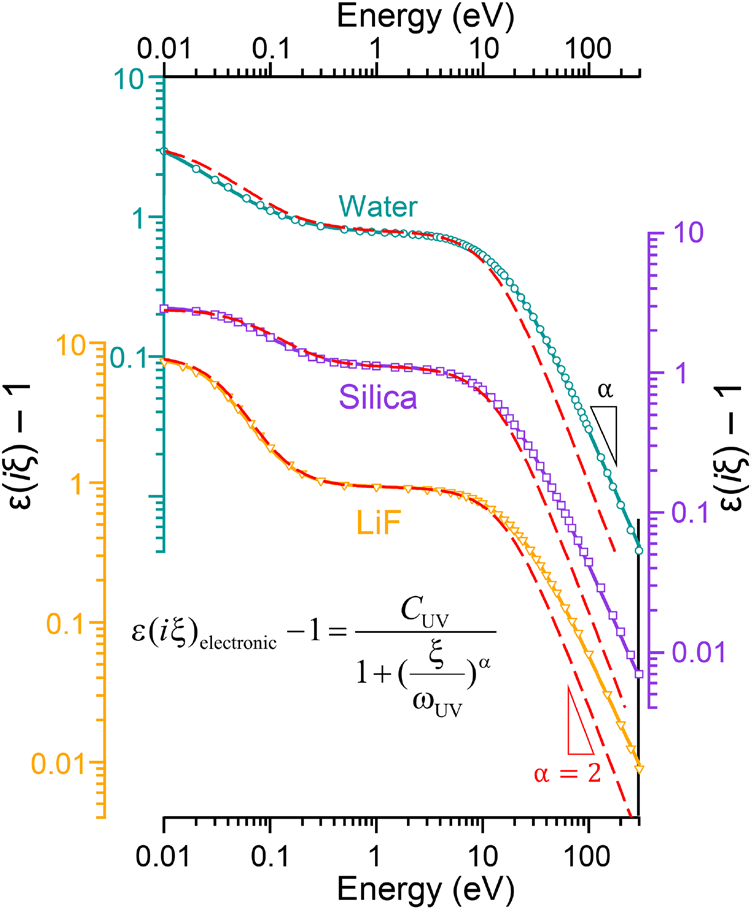
The modified harmonic oscillator model. The graph shows the dielectric functions of water, silica, and LiF at imaginary frequencies. The sets of circular, square, and triangular markers correspond to the values we obtained from numerical transformation of the self-consistent optical constants of the materials (see the Supplementary Materials for more details). The red dashed lines are the estimations based on the single harmonic oscillator model and calculated using the oscillator strengths and frequencies reported by Bergström (*23*) and LeNeveu and Rand (*64*). This model is not consistent with the experimentally derived dielectric functions at frequencies above 10 eV. The solid lines are the estimations produced using the modified harmonic oscillator model, which shows an excellent match with the experimental data.

It has been a common approach to estimate the electronic part of ε(*i*ξ) using a single harmonic oscillator with a negligible damping factor (*23*, *30*)

$$\varepsilon {\left(i\xi \right)}_{\text{electronic}}=1+\frac{C_{\text{UV}}}{1+{\left(\frac{\xi }{\omega_{\text{UV}}}\right)}^{2}}$$ (4)

where *C*_UV_ and ω_UV_ are oscillator’s strength and resonance frequency (often located in the UV region). It is an oversimplification to use one harmonic oscillator to account for the electronic mode of polarization. This approach produces unrealistic approximations. Nevertheless, because of its simplicity, it is widely used (*19*, *20*, *23*–*25*, *38*). This is surprising given that the limitations of this model have been previously addressed (*39*–*43*). In none of the 55 materials, whose optical constants are collected here, was this model able to correctly account for the electronic part of the dielectric functions. The reason being that at high photon energies, Eq. 4 incorrectly predicts that [ε(*i*ξ) − 1] ∼ ω^−2^. This power law behavior is not observed in the current study (Fig. 2). Instead, an analysis of the experimental data shows that [ε(*i*ξ) − 1] ∼ ω^−α^. Therefore, Eq. 4 can be modified to an empirical relationship

$$\varepsilon {\left(i\xi \right)}_{\text{electronic}}=1+\frac{C_{\text{UV}}}{1+{\left(\frac{\xi }{\omega_{\text{UV}}}\right)}^{\alpha }}$$ (5)

**Fig. 3:**
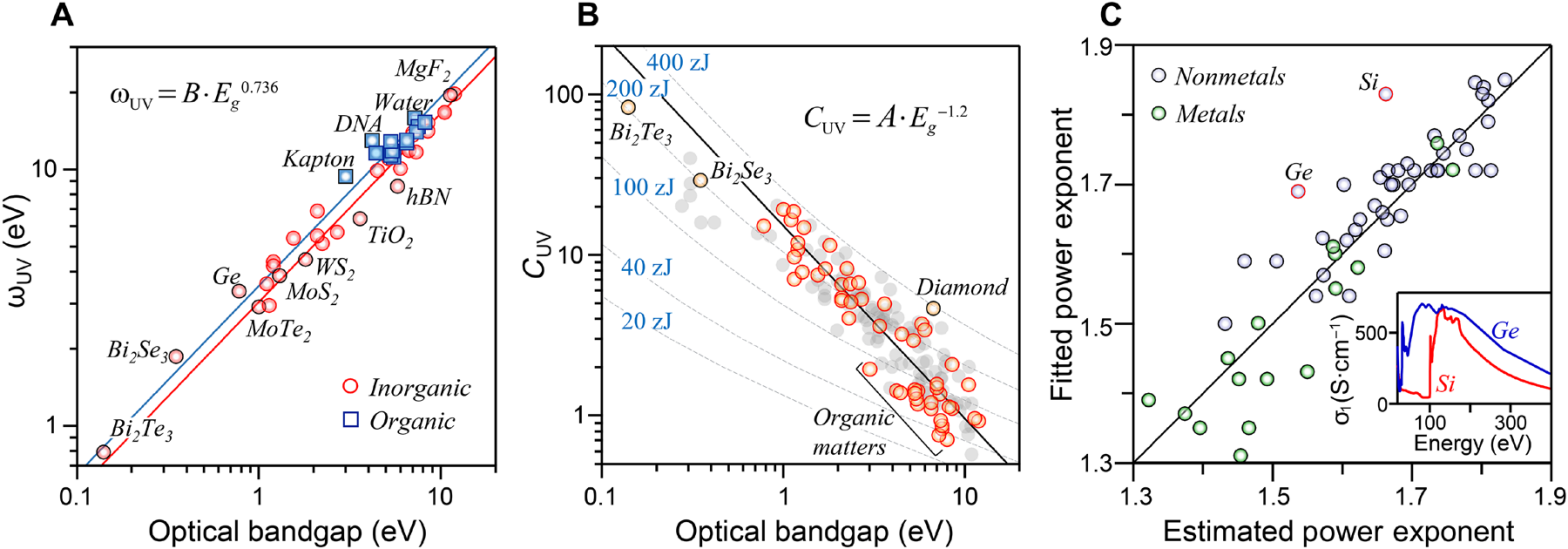
Modeling of the electronic part of dielectric functions of semiconductors and insulators using one modified harmonic oscillator. (**A**) The characteristic frequency (ω_UV_) of different materials versus their optical bandgap. The solid lines are the power law fit to the data with two different prefactors, *B*, for organic (3.55 ± 0.09) and inorganic (3.05 ± 0.06) materials. The power law relationship predicts ω_UV_ over the entire studied range of the bandgap. (**B**) The oscillator strength, *C*_UV_, versus the optical bandgap, *E_g_*. The orange circles are the data on materials whose optical properties are reviewed in detail in the present study. The gray circles are the data on over a hundred other materials reported in two handbooks (*35*, *65*). The inverse correlation between *C*_UV_ and *E_g_* can be estimated using a power law relationship. The contour lines represent the estimated nonretarded Hamaker constants of materials based on the electronic part of their dielectric functions, with *B* = 3.05 and α = 1.7. (**C**) The power exponent in the modified harmonic oscillator model obtained from fitting the experimental dielectric functions against the estimated values using Eq. 7. The violet circles represent the data for semiconductors and insulators, and the green circles represent the data for metals. The line signifies a perfect correlation. The inset shows the real part of the optical conductivity of silicon and germanium in the VUV region where they demonstrate large absorption bands. The deviation in the estimated power exponents for these elements is due to their absorption bands.

Using the modified harmonic oscillator model, it is possible to fit the dielectric functions of all materials reviewed here with high accuracy (Fig. 2), normally with an error of less than 5% (fig. S2). The oscillator strength, *C*_UV_, by definition, is the electronic dielectric constant (ε_∞_) minus 1. For insulators and semiconductors, this is approximated by

$$C_{\text{UV}}=\varepsilon_{\infty }-1={n_{\text{IR}}}^{2}-1$$ (6)

where *n*_IR_ is the refractive index in the near-IR region, provided that there is no strong resonance in this region. The other parameter in the model, ω_UV_, is the characteristic frequency where the main absorption bands are located. In semiconductors and insulators, these bands are necessarily located above the optical bandgap (*E_g_*) of the material. Therefore, it is expected that ω_UV_ positively correlates with *E_g_*. Figure 3A confirms this correlation. Also, one can find that ω_UV_ = *B* · *E_g_*^0.736^, where *B* is an empirical prefactor. This dependence holds for a very wide bandgap range, from 0.14 eV for Bi_2_Te_3_ to 11.9 eV for LiF. It should be noted that *C*_UV_ also scales with *E_g_*, but there is a negative correlation. Figure 3B shows that *C*_UV_ = *A* · *E_g_*^−1.2^ for *E_g_* starting at 0.8 eV, and *A* is another empirical prefactor. This inverse correlation between the refractive indices of materials and their *E_g_* has been known for more than 70 years (*44*). However, a precise prediction of the refractive index solely based on *E_g_* is not possible, and details of the band structure of the materials are required to accurately compute their refractive index (*44*). Nevertheless, to approximate *C*_UV_, it is sufficient to know the refractive indices of materials below *E_g_*. These data are widely measured and tabulated for different materials.

The new parameter in the modified harmonic oscillator used here is the power exponent, α. For all materials studied here, this exponent is always a number between 1 and 2. In the case of semiconductors and insulators, α is smaller for materials containing heavier elements; for instance, it is 1.85 for diamond, 1.73 for Al_2_O_3_, and 1.5 for Bi_2_Te_3_. This observation is anticipated given that the tail of ε(*i*ξ) at high photon energies determines this exponent and knowing that, at high enough photon energies, optical properties of condensed matters can be modeled based on the atomic scattering factors of their constituting elements (*33*). At high photon energies, the power exponent can be approximated as follows (see the Supplementary Materials under section for derivation)

$$\alpha \approx S\cdot \text{log}(\frac{2[1-n]}{A})-0.545$$ (7)

where *n* is a refractive index in the x-ray region (here, 10 keV was chosen) and *S* is an empirical constant equal to −0.36 for inorganic materials and −0.367 for organic materials. *A* is the empirical prefactor relating the bandgap to *C*_UV_, *A* = *C*_UV_ · *E_g_*^1.2^. In Figure 3C, the values predicted by Eq. 7 are compared to the experimental ones where the estimated refractive indices of materials at 10 keV are used (*33*). On the basis of Eq. 7, one can calculate the power exponent for different materials using minimum data input. The large deviation of silicon or germanium from the prediction from Eq. 7 is due to the large absorption bands in the 30- to 300-eV region of the absorption spectrum of these elements (Fig. 3C and figs. S39 and S54). This deviation arises from the fact that experimental ε(*i*ξ) values are fitted around the same energy range to get the power exponents. It should be noted that the modified harmonic oscillator model is an approximation and that the tail of ε(*i*ξ) − 1 at high photon energies does not necessarily decay in a power law behavior with a single exponent.

On the basis of the above analysis, to calculate the electronic part of ε(*i*ξ), one just needs to have ε_∞_, the optical bandgap, density, and the chemical composition of the material. For semiconductors and insulators, these inputs are mostly available. For these two classes of materials, the polarizations at lower photon energies (below IR) usually have a small contribution to the magnitude of Casimir-vdW forces (*30*). Even these modes of polarization can be modeled using the modified harmonic oscillator model. We illustrate this matter with some examples. If, for instance, there is a sharp absorption band in the IR region, then a simple harmonic oscillator is enough to model the response of the matter. However, if there are multiple absorption bands, especially with large damping factors, then the modified harmonic oscillator model should be used. For bands scattered over a broad photon energy range, the power exponent starts to deviate more from the ideal value, i.e., 2. At very low photon energies, Eq. 5 becomes equivalent to the Cole-Cole equation (*45*), which is capable of fitting the experimental data of dielectric function of polar materials. Therefore, for absorption bands in the IR region and below, Eq. 5 relies on fitting of the pre-existing optical data. However, for electronic polarization, the parameters in Eq. 5 can be predicted from the physical properties of materials without the need to access the optical constants. For all semiconductors and insulators studied here, a maximum number of three modified harmonic oscillators were sufficient to model the dielectric function from zero frequency to the x-ray region (see the Supplementary Materials). These analytical forms of dielectric functions showed good agreement with numerical data (fig. S2) and might be used for force calculations at the expense of minor loss of accuracy. However, the main advantage of Eq. 5 is its capability to predict the electronic part of ε(*i*ξ) for materials with unknown optical constants at the UV region.

### Metals and the magnitude of Casimir-vdW forces

The optical constants of metals at low photon energies are largely dominated by free-charge carriers (*27*). Classically, the Drude model is used to compute the optical response of metals (at least in elemental metals) using two parameters: the plasma frequency ω*_p_* and the damping frequency γ (*46*). At higher photon energies (normally above ω*_p_*), the contribution of absorption bands from interband transitions becomes substantial. To what extent these two parts of dielectric functions of metals affect Casimir-vdW forces has not been explicitly addressed. Quite interestingly, though, some researchers tend to take into account just the Drude band when calculating Casimir-vdW forces, even though this approximation can be drastically incorrect (*19*, *20*).

**Fig. 4:**
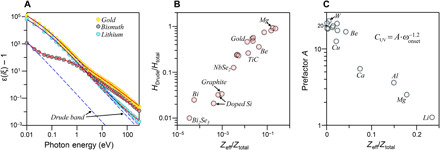
The analysis of the magnitude of Casimir-vdW forces in metals. (**A**) Dielectric functions of lithium, gold, and bismuth in imaginary frequencies. The sets of circular markers represent data derived from numerical calculations. The dashed lines are the contribution of the Drude band calculated from ω*_p_* and γ for each element. The solid red lines are the fits using a Drude band and one modified harmonic oscillator. (**B**) The ratio between the magnitude of nonretarded Hamaker constants of metals derived from just Drude band (*H*_Drude_) and full spectrum of the dielectric functions (*H*_total_) of metals versus the ratio of effective number of free carriers (*Z*_eff_) to total number of electrons in the system. *Z*_eff_ values are obtained from ω*_p_* of each metal. The ω*_p_* is computed by fitting the experimental data at low photon energies. The parameters of the Drude band of doped silicon are based on the report by Barta (*66*). (**C**) The relation between the prefactor *A* and the ratio of the effective number of free carriers (*Z*_eff_) to the total number of electrons in different metals. The values for *A* are computed from fitted parameters for different metals based on the relations given in the main text.

An analysis of ε(*i*ξ) derived from the full spectrum of optical constants of different metals showed that, in the case of light metals such as lithium or aluminum, the Drude band is dominating up to the VUV region. We also found out that for most metals, ε(*i*ξ) can be estimated summing the Drude band and the modified harmonic oscillator to account for the contribution of the interband transitions. Figure 4A represents ε(*i*ξ) for three different elements: lithium, gold, and bismuth. Bismuth is a semimetal with a charge carrier concentration of about five orders of magnitude lower than the charge carrier concentration of the other two metals (*47*). The Drude band accounts for more than half of ε(*i*ξ) up to 42, 5, and 0.04 eV for lithium, gold, and bismuth, respectively. This suggests that, for many metals, omitting the absorption bands from the interband transitions is inaccurate.

To quantify the impact of the Drude band (free carriers), nonretarded Hamaker constants (*H*, a measure of the magnitude of Casimir-vdW forces) are calculated for two scenarios: first, when the full spectrum of the optical constants of metals is used (*H*_total_) and, second, when just the Drude band is used (*H*_Drude_). The ratio $\frac{H_{\text{Drude}}}{H_{\text{total}}}$ is significantly different for various metals, ranging from 92.1% for Mg to 22.9% for Ni. For bismuth, a semimetal, it drops to 2.5%. From ω*_p_*, one can derive the effective carriers’ density $Z_{\text{eff}}=\frac{{\omega_{p}}^{2}\varepsilon_{0}m_{e}}{e^{2}}$ (please note that this is not the real carriers’ density as the value of the carrier’s effective mass is required to compute that quantity) (*27*). The ratio $\frac{H_{\text{Drude}}}{H_{\text{total}}}$ shows a correlation with the ratio of effective carriers’ density to electrons’ density ($Z_{\text{total}}=\frac{N_{A}\rho Z}{M_{w}}$, where *Z* is the effective atomic number). This correlation holds true even for semimetals and doped semiconductors (Fig. 4B). Our analysis indicates that there are only few metals whose Drude bands are strong enough to account for most of the vdW-Casimir forces, at least at short distances. For most metals, the contribution of the interband transitions is essential for an accurate calculation of forces (*46*). Also, it is unlikely that, just by doping semiconductors, the magnitude of Casimir forces (at least in short distances) changes significantly, except for those materials containing light elements, such as graphite and likely silicon. This is consistent with the observation of Chen *et al*. (*48*). After significant silicon doping, they could measure up to 10% increase in Casimir forces, at a distance of around 100 nm between silicon and gold. In addition, the observations of Lannuzzi *et al*. (*49*) can be used to support the above statements. They did not detect any noticeable change in the magnitude of Casimir-vdW forces between gold and a hydrogen-switchable mirror upon hydrogenation. If the contribution of the Drude band is small in a metal, then nulling it should not have a notable effect on the magnitude of Casimir-vdW forces. However, the impact of the protective metal (Pd) in this system might affect the magnitude of the force because the carrier concentrations in those hydrogen-switchable mirrors are comparable to typical elemental metals (*50*).

The contribution of interband transitions to dielectric functions of metals can be estimated using the modified harmonic oscillator. While the experimental data can be fitted successfully using the modified harmonic oscillator, it is a rather arbitrary model for metals. However, if we treat metals without the Drude band the same way we treat semiconductors, then upon using the protocol explained above, it is possible to approximate the power exponent of the modified harmonic oscillator model. These estimations unexpectedly follow the same trend observed for semiconductors and insulators (Fig. 3C). Also, ω_UV_ correlates with the onset of interband transitions (ω_onset_) in most metals studied here. This correlation is analogous to the dependence of ω_UV_ on the optical bandgaps in semiconductors and insulators, which makes it possible to estimate ω_UV_; $\omega_{\text{UV}}=3.05\times \omega_{\text{onset}}^{0.736}$. Extending this analogy even further, and assuming that $C_{\text{UV}}=A\cdot \omega_{\text{onset}}^{-1.2}$, the prefactor *A* derived from fitting the experimental dielectric functions shows an inverse correlation with $\frac{Z_{\text{eff}}}{Z_{\text{total}}}$ (Fig. 4C) in the case of 12 metals studied here. When $\frac{Z_{\text{eff}}}{Z_{\text{total}}}$ is below 0.025, the prefactor *A* is 20 ± 2.1, similar to many semiconductors (see Fig. 3B). However, accurate estimation of ω_onset_ is not always possible with the limited data for metals.

### Theory versus experiments

The presence of Casimir-vdW forces between materials has been demonstrated experimentally by numerous techniques (*24*, *25*, *46*, *51*). Among them is the atomic force microscopy (AFM) technique, which makes it possible to measure the interaction between almost any two materials in air or in a wide range of liquids (*8*, *51*). While Lifshitz’s theory has been successfully used to predict the measured forces between metals (or semiconductors) in air (*46*), the success is limited when these measurements are done in liquids (*41*, *51*). Given that Casimir-vdW forces between materials 1 and 3 interacting through medium 2 depend on

$$F_{\text{vdW}}∼(\frac{\varepsilon_{1}-\varepsilon_{2}}{\varepsilon_{1}+\varepsilon_{2}})(\frac{\varepsilon_{3}-\varepsilon_{2}}{\varepsilon_{3}+\varepsilon_{2}})$$ (8)

these forces are very sensitive to the accuracy of ε(*i*ξ) when the dielectric function of the medium is close to that of the interacting materials. For instance, in the case of mica interacting through water, the estimated nonretarded Hamaker constants vary between 19.8 and 2.6 zJ (*42*).

**Fig. 5:**
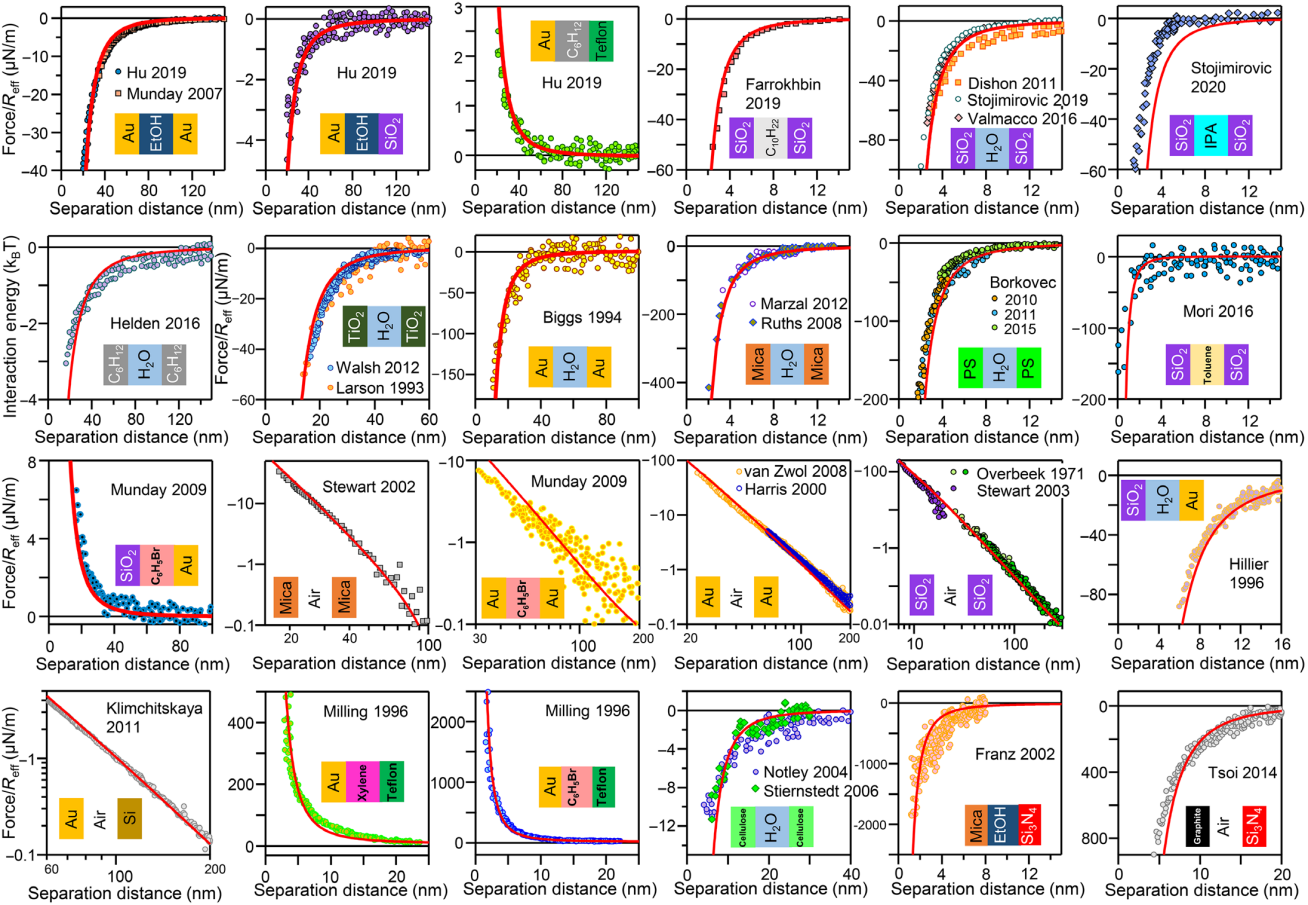
Comparison between the measured and calculated Casimir-vdW forces. The data presented in the form of multicolored dots are the measurements reported in 30 different research papers during the past half-century, involving 19 different materials. The red lines are calculations used in this study. For those materials, whose optical properties were not collected in this study, their dielectric functions are approximated based on their optical bandgaps, refractive indices, chemical composition, and density according to the protocol explained in the text. The calculated Hamaker function for each set of measurements and references are given in the Supplementary Materials (fig. S8). Note that some of the forces are presented in log-log scale.

Figure 5 shows the compilation of Casimir-vdW forces among 24 different combinations of materials measured in the past half-century. We calculated the forces in each system using Lifshitz’s theory (see Methods) and dielectric functions derived from the self-consistent optical constants of these materials. In the case of materials whose optical properties are not studied here, the dielectric functions are approximated by using the modified harmonic oscillator model (table S1). It should be emphasized that no fitting parameters are used for these calculations.

## DISCUSSION

Our as-calculated Casimir-vdW forces show a remarkable agreement with the experimental data in all presented systems. This agreement spans separation distances from as small as 2 nm to much larger separation distances of 300 nm. Our successful prediction of the experimental data is due to the accuracy of our as-calculated dielectric functions. As an example, while the nonretarded Hamaker constant of rutile TiO_2_ across water was calculated to be as low as 19.4 zJ (*25*), here we calculated it to be around 125 zJ. This result agrees with the measured Hamaker constant reported in two independent experiments (*52*, *53*). The agreement between computed and measured forces also confirms that our empirical modified harmonic oscillator model, developed in this study, can reliably predict the dielectric function of materials even in instances where the full spectra of their optical constants are not available (table S1). One such case was regarding the large group of soft matters consisting of polymers, proteins, and lipid membranes, which were successfully modeled based on the limited data available.

Some deviations, however, were observed at small separation distances. These deviations may partly arise because of the sub-nanometer error in the estimation of the separation distances in AFM measurements (*54*). However, the main source of these deviations is probably the surface roughness (*55*, *56*). This effect is more evident when the separation distance is smaller than the surface roughness (*57*). While considerable efforts have been made to address the impact of roughness on surface forces, these analyses are not always conclusive (*56*–*58*).

In conclusion, by evaluating a large set of experimentally measured self-consistent dielectric functions of different materials, we introduced a novel empirical modified harmonic oscillator model in the present work. This improved model can predict the electronic polarization of semiconductors and insulators with only four inputs: the electronic dielectric constant, optical bandgap, density, and the chemical composition. The estimated forces calculated based on our improved model showed excellent agreement with the experimentally measured Casimir-vdW forces. In the case of metals, our analysis demonstrated that the role of interband transitions on the magnitude of vdW-Casimir forces becomes critical once the ratio of charge carriers to total electrons in the systems becomes small.

## METHODS

The imaginary part of the dielectric function of materials studied in this paper was compiled from experimentally measured optical constants from different resources (details are presented in the Supplementary Materials; see figs. S9 to S63). The self-consistency of these data for each material was evaluated by calculating the real part of the refractive index using K-K relation as follows

$$\varepsilon_{1}(\omega )=1+\frac{2}{\pi }\int_{0}^{\infty }\frac{\varepsilon_{2}(x)\cdot x-\varepsilon_{2}(\omega )\cdot \omega }{x^{2}-\omega^{2}}\mathrm{dx}$$ (13)

The real part of the refractive index is then derived and compared to the experimentally measured values in the visible photon energies where the data are available with high precision (*34*, *35*). An acceptable limit was defined based on the experimental error of the reported refractive indices in the visible range (fig. S1). In addition, dielectric constants calculated from these data were mostly in agreement with the tabulated values. Also, the effective number of electrons is calculated using the following f-sum rule (*27*)

$$n_{\text{eff}}=\frac{2M_{w}\varepsilon_{0}m_{e}}{\pi \rho N_{A}e^{2}\hbar^{2}}\int_{0}^{\infty }\varepsilon_{2}(x)\cdot \mathrm{xdx}$$ (14)

The accuracy of the calculated *n*_eff_ was better than 2% compared to the nominal value. In cases where multiple reports were available on optical constants, we selected datasets that resulted in both accurate estimation of refractive index and *n*_eff_. Occasionally, we readjusted the intensities of absorption bands to obtain realistic refractive indices and *n*_eff_. Overall, errors in measuring the intensity of the absorption bands are more common than the position, as in many experimental techniques, including reflectometry or electron energy loss spectroscopy, the absolute value of optical constants depends on the internal calibration of the instrument and/or postprocessing of raw data (*59*, *60*).

To calculate Casimir-vdW forces, Hamaker functions of materials 1 and 3 interacting across medium 2 were calculated using Lifshitz’s theory as follows (*30*)

$$H_{123}(l)=\frac{3k_{B}T}{2}{\sum_{n=0}^{\infty }}^{'}R_{n}(l)\sum_{s=1}^{\infty }\frac{{\left(\Delta_{12}\Delta_{32}\right)}^{s}}{s^{3}}$$ (15)

where *k_B_T* is the thermal energy and

$$\Delta_{\mathrm{ij}}=\frac{\varepsilon_{i}(i\xi_{n})-\varepsilon_{j}(i\xi_{n})}{\varepsilon_{i}(i\xi_{n})+\varepsilon_{j}(i\xi_{n})}$$ (16)

For anisotropic materials, $\varepsilon_{i}=\sqrt{\varepsilon_{⊥}\varepsilon_{∥}}$. This is a reasonable estimation at short distances and when the principle axes are perpendicular to the interface (*30*, *61*). Also

$$\varepsilon (i\xi )=1+\frac{2}{\pi }\int_{0}^{\infty }\frac{\varepsilon_{2}(x)\cdot x}{x^{2}+\xi^{2}}\mathrm{dx}$$ (17)

$$\xi_{n}=2\pi \frac{{\mathrm{nk}}_{B}T}{\hbar }$$ (18)

$$R_{n}(l)=(1+\frac{2l\xi_{n}\sqrt{\varepsilon_{3}(i\xi_{n})}}{c})e^{-\frac{2l\xi_{n}\sqrt{\varepsilon_{3}(i\xi_{n})}}{c}}\;\text{for}\;n\geq 1$$ (19)

$$R_{n}(l)=(1+2l\kappa )e^{-2l\kappa }\;\text{for}\;n=0$$ (20)

where *l*, *c*, and κ^−1^ are separation distance, speed of light, and Debye length, respectively. The prime in the first summation indicates that the zero-term frequency should be divided by 2. Note that the zero-term frequency is normally small compared to other terms of Eq. 15 or is largely screened by the presence of salts in medium, according to Eq. 20. Only in the case of cellulose-water-cellulose (in Fig. 5) was this term large enough as the measurements were performed at low ionic strength (*62*, *63*). In this case, κ^−1^ is assumed to be 10 nm. The normalized force to effective radius (*R*_eff_) is then calculated using Derjaguin approximation (*8*)

$$\frac{F_{\text{vdW}}(l)}{R_{\text{ef}f}}=2\pi \int_{l}^{\infty }\Pi (x)\mathrm{dx}=\int_{l}^{\infty }-\frac{H(x)}{3x^{3}}\mathrm{dx}$$ (21)

Π is the vdW pressure between two plates.

## SUPPLEMENTARY MATERIALS

Supplementary material for this article is available at http://advances.sciencemag.org/cgi/content/full/7/22/eabg2272/DC1
